# The Anaphase-Promoting Complex (APC) ubiquitin ligase affects chemosensory behavior in *C. elegans*

**DOI:** 10.7717/peerj.2013

**Published:** 2016-05-10

**Authors:** Julia Wang, Alexandra K. Jennings, Jennifer R. Kowalski

**Affiliations:** Department of Biological Sciences, Butler University, Indianapolis, IN, United States

**Keywords:** Anaphase-promoting complex, Chemotaxis, Sensory function, *C. elegans*, Ubiquitin ligase

## Abstract

The regulation of fundamental aspects of neurobiological function has been linked to the ubiquitin signaling system (USS), which regulates the degradation and activity of proteins and is catalyzed by E1, E2, and E3 enzymes. The Anaphase-Promoting Complex (APC) is a multi-subunit E3 ubiquitin ligase that controls diverse developmental and signaling processes in post-mitotic neurons; however, potential roles for the APC in sensory function have yet to be explored. In this study, we examined the effect of the APC ubiquitin ligase on chemosensation in *Caenorhabditis elegans* by testing chemotaxis to the volatile odorants, diacetyl, pyrazine, and isoamyl alcohol, to which wild-type worms are attracted. Animals with loss of function mutations in either of two alleles (*g48* and *ye143*) of the gene encoding the APC subunit EMB-27 APC6 showed increased chemotaxis towards diacetyl and pyrazine, odorants sensed by AWA neurons, but exhibited normal chemotaxis to isoamyl alcohol, which is sensed by AWC neurons. The statistically significant increase in chemotaxis in the *emb-27 APC6* mutants suggests that the APC inhibits AWA-mediated chemosensation in *C. elegans*. Increased chemotaxis to pyrazine was also seen with mutants lacking another essential APC subunit, MAT-2 APC1; however, *mat-2 APC1* mutants exhibited wild type responses to diacetyl. The difference in responsiveness of these two APC subunit mutants may be due to differential strength of these hypomorphic alleles or may indicate the presence of functional sub-complexes of the APC at work in this process. These findings are the first evidence for APC-mediated regulation of chemosensation and lay the groundwork for further studies aimed at identifying the expression levels, function, and targets of the APC in specific sensory neurons. Because of the similarity between human and *C. elegans* nervous systems, the role of the APC in sensory neurons may also advance our understanding of human sensory function and disease.

## Introduction

Olfaction is the part of the nervous system responsible for detecting volatile odorants. In humans, the sense of smell is crucial in that it helps in evaluating one’s surroundings, affects the taste of food, and even acts as an early warning sign to environmental hazards. Approximately two million Americans, including a disproportionate number of elderly individuals, are affected by olfactory or gustatory dysfunction (Spielman, 1998). Such disorders may involve either increased or decreased sensory perception and can result from over 200 different causes, including infections, endocrine dysfunction, toxins, a variety of neurologic disorders such as migraines, seizures, head trauma and neurodegenerative diseases, such as Parkinson’s disease, as well as general age-related decline (Quinn, Rossor & Marsden, 1987; Spielman, 1998). Despite the significant reduction in quality of life for individuals with these disorders, surprisingly little research is focused on uncovering the molecular mechanisms underlying sensory system function and dysfunction.

*Caenorhabditis elegans* roundworms represent an ideal system for investigating olfactory biology and many other aspects of nervous system function. *C. elegans* possesses a simple and completely mapped nervous system, which is comprised of exactly 302 neurons (Brenner, 1974; White et al., 1986; Wood, 1988). Despite its simplicity, *C. elegans* displays a number of behaviors similar to those of complex animals; these behaviors include locomotion, feeding, mating, learning, and sensory responses to touch, smell, and taste (De Bono & Maricq, 2005). Thirty-four *C. elegans* neurons (>10% of the nervous system) are involved in sensory function (Bargmann, 2006), and many basic attributes of the olfactory system are conserved between *C. elegans* and humans. Similarities include the use of G protein-coupled seven-pass transmembrane proteins (GPCRs) as odorant receptors, over 500 of which are encoded in the genomes of both organisms, as well as the use of inositol 3-phosphate (IP_3_) and cyclic AMP/GMP (cAMP/cGMP) signaling pathways downstream of those receptors (Bargmann, 2006; Troemel et al., 1995; Vassar, Ngai & Axel, 1993).

*C. elegans* are attracted to a number of volatile odorants at a range of concentrations (Bargmann, Hartwieg & Horvitz, 1993). Although these worms sense chemicals using five pairs of chemosensory neurons (AWA, AWB, AWC, ASH, and ADL), ASH and ADL neurons are primarily involved in sensing water-soluble chemicals (gustation); thus, AWA, AWB, and AWC neurons appear to mediate the majority of responses to volatile chemicals and are considered the major olfactory neuron classes in *C. elegans* (Zhang et al., 2014). These chemosensory neurons are located in one of two amphid sensory organs in the head and possess sensory cilia exposed to the environment, although the complex ciliated endings of the AWA, AWB, and AWC neurons are covered by a sheath cell that prevents direct contact with the external medium (Hart & Chao, 2010; Ward et al., 1975; White et al., 1986). Each chemosensory neuron senses a unique set of odorants; these bind primarily to GPCRs located in the sensory cilia of the neurons to initiate intracellular signaling as described above (Zhang et al., 2014). Such signaling in the sensory neurons leads to the release of neurotransmitters that act on primary interneurons; these cells, in turn, synapse with secondary and command interneurons, which ultimately relay signals to motor neurons to control movement of the worms in response to these sensory cues (Tsalik & Hobert, 2003).

Laser ablation experiments demonstrated that both AWA and AWC sensory neurons mediate attractive chemotaxis responses to certain odorants (Bargmann, Hartwieg & Horvitz, 1993); in contrast, AWB neurons mediate responses to volatile repellants (Troemel, Kimmel & Bargmann, 1997). Attractants sensed by AWC neurons include isoamyl alcohol, trimethylthiazole, benzaldehyde, 2-butanone, and pentanedione; AWA neurons also detect trimethylthiazole, as well as diacetyl and pyrazine (Bargmann, Hartwieg & Horvitz, 1993). Many of these attractive molecules, including isoamyl alcohol, diacetyl, and pyrazine, are byproducts of bacterial metabolic processes; thus, in the wild, the ability to sense these odorants likely assists the worms in locating food sources (Bargmann, 2006). Although several of the components of the basic pathways and proteins that mediate odorant recognition and response are known, receptors for only a few odorants have been identified and the molecular regulation of the signaling pathways that mediate recognition of and responses to these odorants has yet to be fully explored.

One way that the development and function of neurons of all types are controlled is through the ubiquitin signaling system (USS) (DiAntonio & Hicke, 2004; Ding & Shen, 2008; Kowalski & Juo, 2012; Tai & Schuman, 2008; Yi & Ehlers, 2007). Ubiquitin is a 76 amino acid polypeptide that is added as a covalent modification either singly or in various branched chain configurations to lysine residues in target proteins. Ubiquitination of proteins leads to a change in their function or localization or to their degradation in either the lysosome or the 26S proteasome, depending on the nature of the branching (Kulathu & Komander, 2012). The activity of the USS is catalyzed by the sequential activity of E1 activating enzymes, E2 conjugating enzymes, and E3 ubiquitin ligases that are responsible for recognizing and covalently attaching ubiquitin polypeptides to the substrate for ubiquitination (Hershko & Ciechanover, 1998). There are two E1, 40 E2, and over 600 E3 ubiquitin ligases encoded in the human genome; similar numbers are found in *C. elegans* (Baptista, Duarte & Maciel, 2012; Li et al., 2008b).

The USS plays a major role in a number of neuronal processes by maintaining protein homeostasis. Misregulation of the USS is linked to neurological and neurodegenerative disorders, such as Angelman’s syndrome and Parkinson’s disease (Tai & Schuman, 2008; Yi & Ehlers, 2007). Evidence from diverse organisms also suggests the importance of the ubiquitin system in regulating sensory function. For example, ubiquitination and degradation of the Bcl2-associated pro-survival protein Bag-1 is correlated with apoptosis of olfactory neurons, which is required for normal olfactory neuron turnover (Sourisseau et al., 2001). The USS has also been implicated in controlling axon and dendrite outgrowth of sensory neurons, as flies either over- or underexpressing the Angelman’s syndrome-associated ubiquitin ligase Ube3A (*dUb3a*) exhibit reduced terminal dendritic branching of peripheral sensory neurons (Lu et al., 2009). Similarly, olfactory neurons in mice lacking expression of the Mycbp2 ubiquitin ligase fail to project to the dorsal olfactory bulb surface - a defect that appears to be due to the ability of Mycbp2 to regulate expression of the Robo2 axon guidance receptor (James, Key & Beverdam, 2014).

Several recent studies also confirm the importance of the USS in controlling sensory processing or signal transduction and identify ubiquitin ligases involved in this regulation. Cold pain sensation and the function of specific central and afferent pain pathways were found to be reduced in Parkinson’s disease patients carrying mutations in the parkin E3 ligase (Gierthmuhlen et al., 2010), suggesting important roles for the USS in controlling the signaling and/or connectivity of these neurons. Loss of function of the Ring finger ubiquitin ligase RNF170, mutation of which is linked with a rare autosomal-dominant sensory ataxia (ADSA) in humans, causes reduced proprioceptive sensitivity and thermal pain sensing in mice (Kim et al., 2015). These sensory defects correlate with an age-dependent increase in sensory-dependent walking abnormalities and with elevated levels of type-I inositol 1,4,5-trisphosphate receptors (ITPR1), endosome-associated Ca^2+^ channels, in the cerebellum and spinal cord. As RNF170 previously was shown to regulate ubiquitination-dependent degradation of ITPR1, these findings suggest a role for ubiquitin-mediated regulation of sensorimotor coupling (Kim et al., 2015). Finally, a specific role for ubiquitin-mediated regulation of receptor cell sensitivity was recently demonstrated in *C. elegans* as ubiquitination of the mechanosensory channel subunit MEC-4 (which requires the gene encoding the E3 ubiquitin ligase *mbf-1*) was found to regulate MEC-4 abundance and, thus, mechanosensory stimulus-induced currents in the processes of ALM mechanosensory neurons (Chen & Chalfie, 2015). Together, these studies demonstrate that USS activity is critical to sensory system formation and function. Nevertheless, although a handful of ubiquitin ligases that regulate sensory physiology have been identified, much remains to be learned about the potential roles of the hundreds of other ubiquitin ligases and their target proteins in these processes.

One such E3 ubiquitin ligase, the Anaphase-Promoting Complex (APC), is responsible for ubiquitinating multiple target proteins through which it regulates many different neuronal processes (Manchado, Eguren & Malumbres, 2012; Puram & Bonni, 2011). The APC is one of the largest E3 ligase complexes, composed of 11–19 subunits, including two different substrate adaptor subunits, Cdh1 and Cdc20 (Chang et al., 2014; Peters, 2006). These adaptors aid in recognizing specific amino acid sequences (e.g., the Destruction box (D-box), RxxLxxxxN/D/E, or the KEN-box, KENxxxN/D/E) in APC target proteins (Harper, Burton & Solomon, 2002; Peters, 2006). The APC, in conjunction with its E2, then typically adds K11-linked ubiquitin chains that promote substrate degradation (Jin et al., 2008; Kulathu & Komander, 2012; Williamson et al., 2011; Wu et al., 1999); however, multiple mono-ubiquitination of cyclin D1 by the APC has also been shown to promote its proteasomal destruction (Dimova et al., 2012). In addition to its initially described role in controlling various aspects of the eukaryotic cell division cycle, the APC has multiple functions in post-mitotic neurons, regulating processes such as axon outgrowth, dendrite development, neuronal differentiation, synapse development and pre-synaptic specialization (Puram & Bonni, 2011; Wise et al., 2013). The APC also regulates transmission at glutamatergic interneuron synapses (Fu et al., 2011; Juo & Kaplan, 2004; Van Roessel et al., 2004) in worms, flies, and mammalian cells, and controls synaptic *γ*-Aminobutyric acid (GABA) signaling at the *C. elegans* neuromuscular junction (NMJ) to regulate the balance of excitatory to inhibitory transmission needed for muscle contraction (Kowalski et al., 2014). A requirement for the APC in controlling GABA ergic signaling relevant for several processes involved in learning and memory in mammals has also been shown (Kuczera et al., 2011; Li et al., 2008a; Pick, Malumbres & Klann, 2012; Pick et al., 2013).

Although functions of the APC have been established in post-mitotic interneurons and at the NMJ, whether the APC also acts in sensory neurons remains unknown. A recent study investigating mechanisms to enhance regeneration of dorsal root ganglion (DRG) neurons in mice found that overexpression of a mutant version of the Id2 protein lacking the D-box motif recognized by the APC enhanced neurite formation following injury, suggesting a role for the APC in sensory neurons in this system (Yu et al., 2011). Given the role of the APC in numerous other neuronal processes, we hypothesized that the APC also regulates chemosensory function in *C. elegans*. Here, we used chemotaxis of *emb-27 APC6* and *mat-2 APC1* loss of function mutants to diacetyl, pyrazine, and isoamyl alcohol to show that specific subunits of the APC are required for normal chemosensation in *C. elegans*.

## Materials and Methods

### Chemicals

Sodium azide, 95% ethanol, isoamyl alcohol (1:10,000 dilution), diacetyl (1:5000 dilution), and pyrazine (1mg/mL) (all from Sigma-Aldrich). All odorants were diluted in 95% ethanol.

### Strain maintenance

Worm strains used in this study include wild type (N2), *odr-7(ky4)*, *emb-27(g48ts), emb-27(ye143ts),* and *mat-2(ax102ts)*. The *odr-7(ky4)* mutants, which have defects in AWA neuron fate, were used as a positive control in all experiments, as they are known to exhibit reduced chemotaxis to AWA neuron-specific odorants, including diacetyl and pyrazine (Sengupta, Colbert & Bargmann, 1994). The other strains carry temperature-sensitive point mutations in the genes encoding the respective subunit of the APC (Davis et al., 2002; Golden et al., 2000). These mutations allow the worms to grow normally at 15 °C but cause a loss of function at 25 °C due to misfolding of the mutant protein.

All *C. elegans* strains were grown at 15 °C on petri plates containing NGM agar with the *E. coli* strain OP50 as a food source (Brenner, 1974). When a petri plate became crowded, three L4 worms were picked onto a new plate. To prepare the worms for the chemotaxis assays, worms were grown until the plate was filled with egg-laden adults. Twelve egg-laden adults were picked onto freshly spotted plates. The number of plates depended on the number of attractants being tested. These adults were allowed to crawl around for two hours to lay eggs. After 2 h, the adults were removed from the plates, leaving only eggs. The eggs were incubated at 15 °C for four days. On the fifth day, worms had reached the fourth larval (L4) stage and were incubated at 25 °C for 20 h to induce loss of function in the APC. Wild type and *odr-7* mutants were also synchronized and exposed to temperature shift along with the APC mutants.

**Figure 1:**
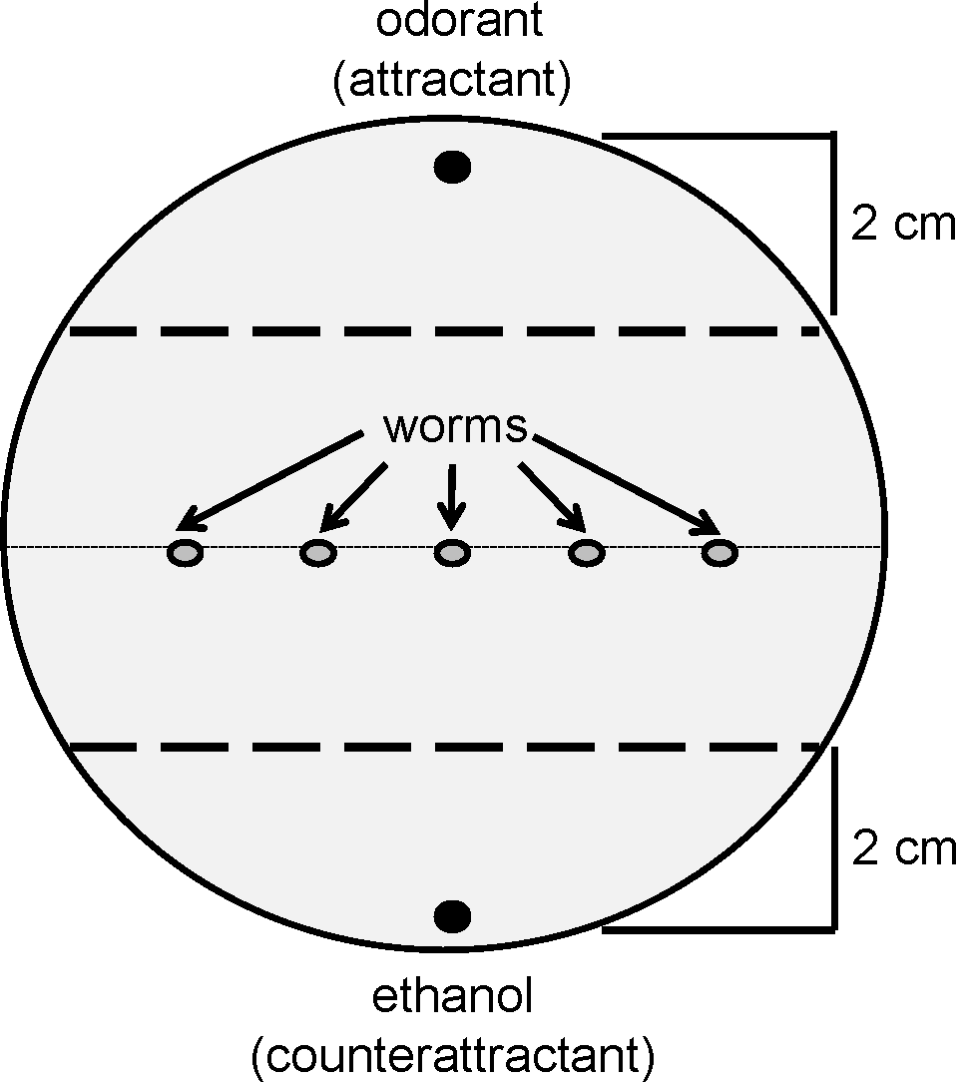
Diagram of a chemotaxis assay plate. The odorant and sodium azide were placed on the “odorant” dot while ethanol (vehicle) and sodium azide were placed on the “ethanol” dot on opposite sides of a 10 cm NGM agar plate. Each dot was 0.5 cm from the edge of the plate. Two lines were drawn (large dashes), one 2 cm from the odorant and one 2 cm from the ethanol, and a center dividing line (small dashes) was drawn between them down the middle of the plate. The worms then were placed in five drops on the center dividing line. At the end of the assay period, worms beyond the 2 cm line on the odorant side or beyond the 2 cm line on the ethanol side were deemed to be at the attractant or the counterattractant respectively. The chemotaxis index was calculated for each plate as described in ‘Materials and Methods.’

### Chemotaxis assays

Chemotaxis assays were performed as previously described (Bargmann, Hartwieg & Horvitz, 1993), but with the following modifications. Four to six days before the assay, 10 cm plates, each containing 20 mL of NGM agar, were prepared and stored at room temperature. Prior to the start of the assay, the 10 cm plates were marked with a line through the center and two marks on each side of the line 0.5 cm from the edge of the agar. One mark was labeled for the odorant (attractant), and the other for the ethanol (counterattractant) (Fig. 1). Two lines were drawn, one 2 cm from the attractant and the other 2 cm from the counterattractant. Next, 10 µL of 1 M sodium azide were placed on each mark. The azide was allowed to dry, then 10 µL of the odorant were placed on the odorant mark and 10 µl of 95% ethanol were placed on the ethanol mark. This was done for each odorant (isoamyl alcohol @ 1:10,000 dilution, diacetyl @ 1:5,000 dilution, pyrazine @ 1 mg/ml) on four separate plates for each strain of worms to be tested. The adult worms were then washed off the synchronized growth plates with 5 mL of M9 buffer into a conical tube. After the worms settled, the M9 buffer was drawn off, and the worms were washed twice more with M9 buffer. The worms then were washed once with water, and the water was removed until only 150–200 µL of solution remained. A drop containing 4 µL of the resuspended worm solution was placed on each 10 cm plate at the center of the central dividing line. This was repeated five times for a total of 20 µL of worm solution, so that the five drops were evenly placed on the central dividing line of each plate (Fig. 1). The worm solution was flicked before each drop was pipetted to ensure that the worms were evenly dispersed in the solution. A kimwipe was used to remove excess liquid from the worm droplet to ensure synchronous drying of the liquid on each plate. Worms were allowed to migrate on the assay plates for 1 h at room temperature. Because of the sodium azide, after the worms reached the attractant or counterattractant, they became paralyzed; this was done to prevent worms from crawling away from the odorants due to adaptation to the stimulus. After 1 h, the number of worms past the attractant line, the number past the counterattractant line, and the total number of worms on the plate were recorded. The chemotaxis index for each plate was calculated according to the following formula:

\begin{eqnarray*}\text{Chemotaxis index}= \frac{(#\text{at attractant})-(#\text{at counter attractant})}{\text{total}#\text{on plate}} . \end{eqnarray*}

### Concentration/time curve assays

Pyrazine was serially diluted to concentrations of 0.5 mg/mL and 0.1 mg/mL from a 1mg/ml stock in 95% ethanol. These dilutions were used as odorants in chemotaxis assays with ethanol as the counterattractant. Chemotaxis data were collected for wild type and *emb-27(g48)* strains, and chemotaxis indices were calculated every 30 min for 2 h for worms at each concentration.

### Statistical analysis

The mean chemotaxis index and standard deviation for each strain were calculated from the independent experiments with each odorant performed on multiple days. For all experiments, the total number of worms per assay plate ranged from 45 to 185 and the chemotaxis index of wild type control worms was above 0.4 for all odorants tested at the standard concentrations. (This cutoff was not used for pyrazine tests at 0.5 and 0.1 mg/ml, as we expected wild type worms to show lower chemotaxis indices at these concentrations). Statistical analyses were performed using the JMP 12 software program and all comparisons were made between individual mutant strains and wild type (N2) worms for each odorant. First, the distribution of each dataset was tested for normality using a Shapiro Wilk Goodness of Fit test. For comparisons in which at least one of the datasets showed a non-normal distribution, a non-parametric Wilcoxon test was used to determine statistical significance. For comparisons in which both datasets were normally distributed, an *F* test for equality of variance was performed, followed by a two-tailed unpaired *t*-test assuming either equal or unequal variance, in accordance with the *F* test result. The *α*-level for all tests was 5%.

## Results

The APC is a regulator of diverse aspects of neuronal function, including neuronal survival, axon and dendrite development, synapse formation, and synaptic transmission at both glutamatergic and GABAergic synapses. Here, we tested whether the APC is also involved in chemosensory function by assessing the ability of *emb-27 APC6* and *mat-2 APC1* loss of function *C. elegans* mutants to chemotax to several volatile attractants—isoamyl alcohol, diacetyl, and pyrazine.

**Figure 2:**
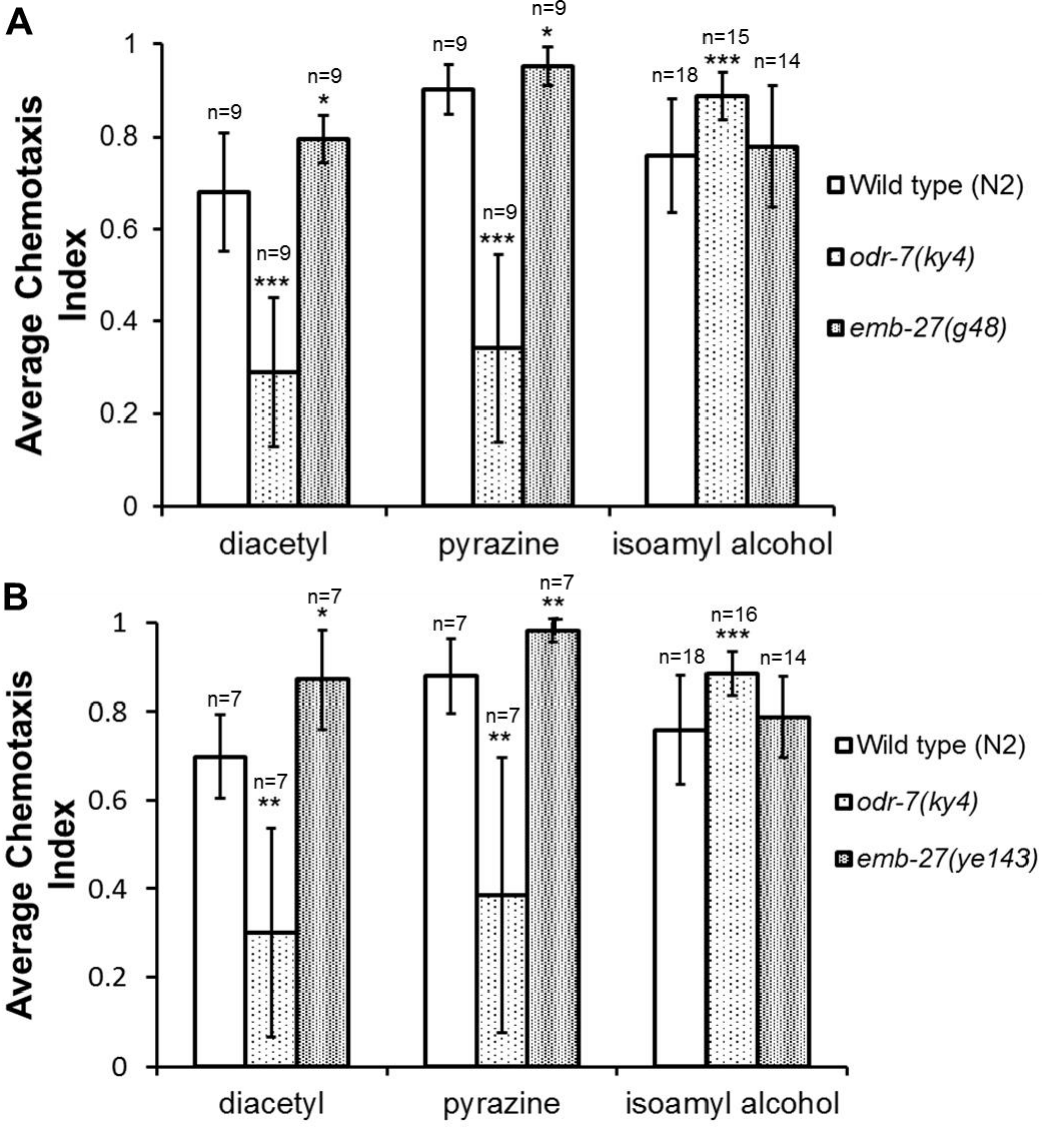
*emb-27 APC6* loss of function mutants show increased chemotaxis towards AWA neuron-specific odorants. Wild type, *odr-7(ky4)*, and (A) *emb-27(g48)* or (B) *emb-27(ye143)* adult worms were placed, following a 20 h temperature shift at the L4 stage, onto each chemotaxis plate along with diacetyl, pyrazine, or isoamyl alcohol as the attractant and ethanol as the counterattractant. After one hour at room temperature, the number of worms at the attractant, the number at the counterattractant, and the total worms on the plate were recorded and chemotaxis indices calculated. The mean value and standard deviation error bars are shown (*n* = 7–18 plates per treatment group, as indicated on the graph). ^∗^*p* < 0.05, ^∗∗^*p* ≤ 0.01, ^∗∗∗^*p* ≤ 0.001, compared to wild type. All values belong to experimental groups and controls tested in parallel.

**Figure 3:**
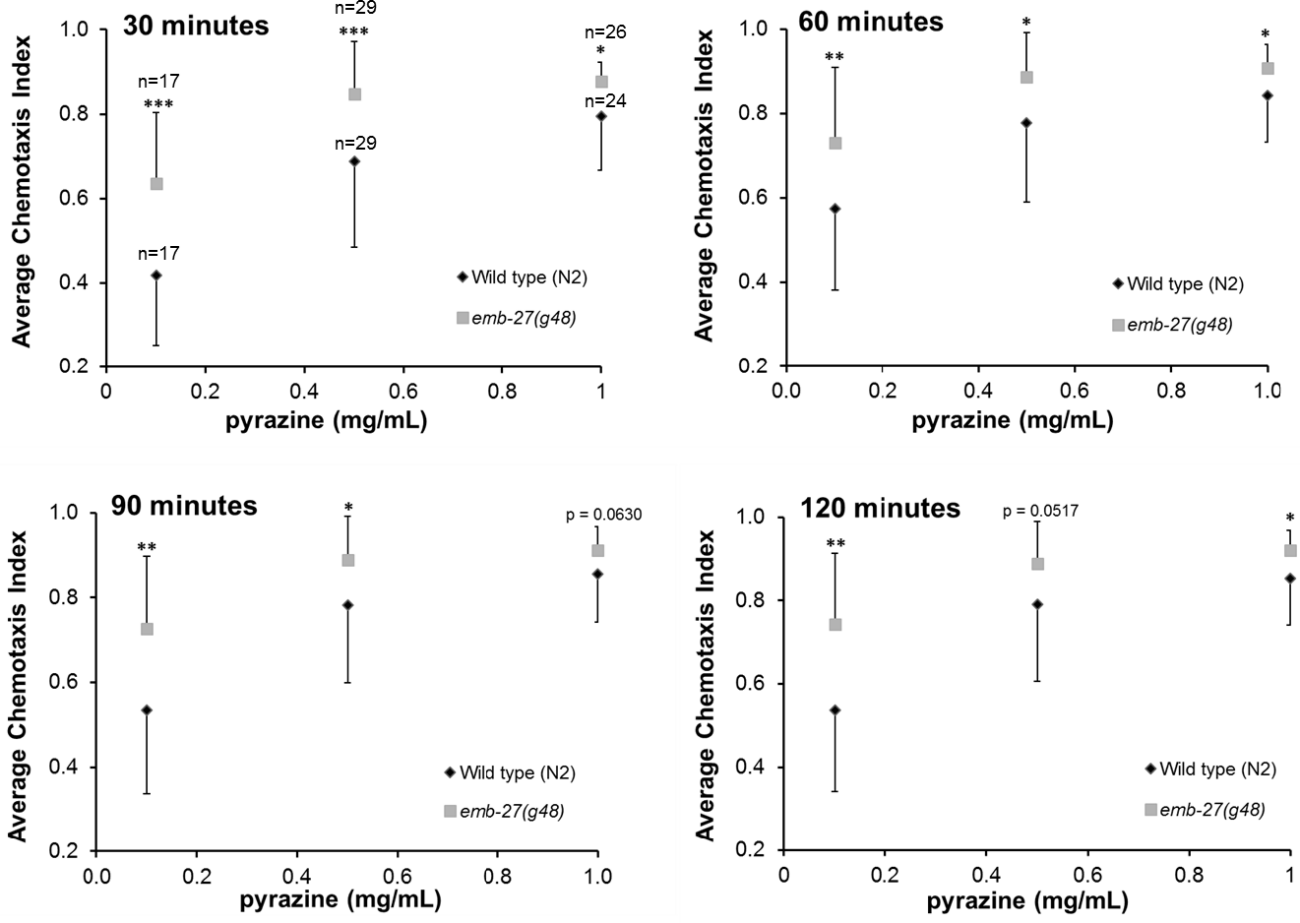
*emb-27(g48) APC6* loss of function mutants show increased chemotaxis to pyrazine across multiple timepoints and concentrations. Wild type and *emb-27(g48)* mutant animals were placed, following a 20 h temperature shift at the L4 stage, onto chemotaxis plates with 0.1 mg/mL, 0.5 mg/mL or 1 mg/mL pyrazine as the attractant and ethanol as the counterattractant. Every 30 min for 2 h, the number of worms at the attractant, the number at the counterattractant, and the total worms on the plate were recorded and chemotaxis indices calculated. The mean values at each concentration were computed for each timepoint and standard deviation error bars are shown (*n* = 17–29 plates per treatment group, as indicated on the graph). Replicate values are included only at 30 minutes since the same plates were counted at 30, 60, 90, and 120 min. ^∗^*p* < 0.05, ^∗∗^*p* ≤ 0.01, ^∗∗∗^*p* ≤ 0.001, compared to wild type.

**Figure 4:**
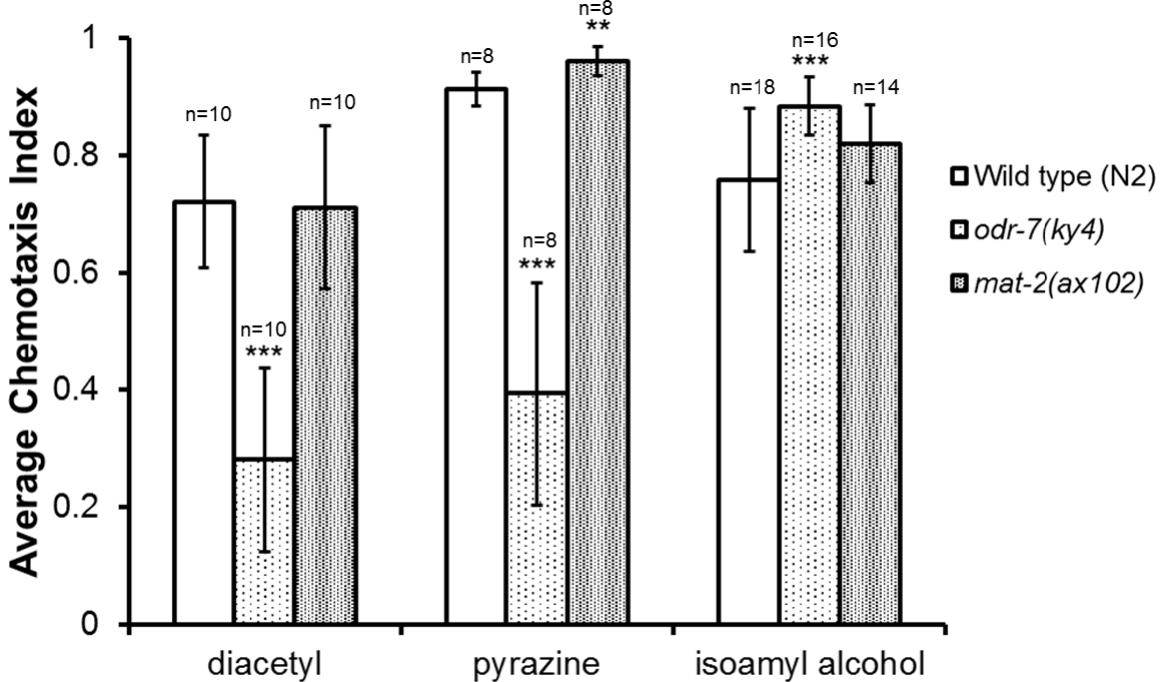
*mat-2(ax102) APC1* loss of function mutants show increased chemotaxis towards pyrazine but not diacetyl or isoamyl alcohol. Wild type, *odr-7(ky4)*, *and mat-2(ax102)* strains were placed, following a 20 h temperature shift at the L4 stage, onto chemotaxis plates with diacetyl, pyrazine, or isoamyl alcohol as the attractant and ethanol as the counterattractant. After one hour at room temperature, the number of worms at the attractant, the number at the counterattractant, and the total worms on the plate were recorded and chemotaxis indices calculated. The mean value and standard deviation error bars are shown (*n* = 8–18 plates per treatment group, as indicated on the graph). ^∗∗^*p* ≤ 0.01, ^∗∗∗^*p* ≤ 0.001, compared to wild type. All values belong to experimental groups and controls tested in parallel.

Wild type worms showed attraction to all three attractants, as previously described (Bargmann, Hartwieg & Horvitz, 1993) (Figs. 2–4). The *odr-7(ky4)* mutants, the positive control, showed reduced chemotaxis to pyrazine and diacetyl (Figs. 2 and 4) compared to wild type worms (*p* ≤ 0.01), as expected (Sengupta, Colbert & Bargmann, 1994), whereas their response to isoamyl alcohol was unimpaired and even slightly increased (*p* ≤ 0.001) (Figs. 2 and 4). We next tested temperature sensitive (ts) APC mutants carrying either of two loss of function alleles of the *emb-27 APC6* gene, *g48ts* and *ye143ts; emb-27 APC6* encodes an essential subunit of the APC whose loss of function is required for normal glutamatergic and GABAergic signaling in *C. elegans* (Juo & Kaplan, 2004; Kowalski et al., 2014). Both the *emb-27(g48)* and *emb-27(ye143)* mutants responded normally to isoamyl alcohol (Fig. 2) but exhibited a statistically significant increase in chemotaxis to diacetyl (16.7% increase, *p* = 0.032 and 25.2% increase, *p* = 0.025, respectively) and pyrazine (5.7% increase, *p* = 0.041 and 11.5% increase, *p* = 0.0039, respectively) compared to wild type worms.

Additionally, we performed concentration–time curves of the responses of wild type and *emb-27(g48)* mutant worms to pyrazine. The *emb-27(g48)* mutants showed increased chemotaxis to pyrazine at every concentration over a ten-fold range (0.1–1 mg/ml) at every time interval tested (30 min–2 h) in comparison to wild type worms (Fig. 3). The increased chemotaxis indices were statistically significant at every timepoint for each of the three pyrazine concentrations with the exception of 90 min at 1 mg/ml (*p* = 0.0630) and at 120 min at 0.5 mg/ml (*p* = 0.0517), which still showed borderline significance. Thus, although the differences begin to equilibrate slightly at later timepoints, in comparison to wild type animals, the *emb-27(g48)* mutants exhibited a consistent, notable increase in chemotaxis across all concentrations of pyrazine over a two hour period. While the chemotaxis difference between the *emb-27* (*g48*) mutants and wild type worms we measured at 1 mg/ml was small but reproducible (5–10%, Figs. 2–3), the difference in chemotaxis between these strains increased markedly at the lower pyrazine concentrations; *emb-27* (*g48*) animals exhibited a 12–23% increase at 0.5 mg/ml and 37–50% increases at 0.1mg/ml relative to wild type controls (Fig. 3).

Finally, we tested the effect of the APC complex protein MAT-2 APC1 on chemosensation toward diacetyl and pyrazine in order to determine if the increased chemotaxis seen with the *emb-27 APC6* mutants was specific to the EMB-27 APC6 protein or whether the full complex may be involved, as we predicted. Interestingly, *mat-2(ax102) APC1* mutant worms showed a statistically significant increase in chemotaxis toward pyrazine compared to wild type worms (*p* = 0.0028) but exhibited wild type responses to diacetyl and isoamyl alcohol (*p* > 0.05) (Fig. 4). Together, these data indicate a role for several APC subunits in negatively regulating aspects of chemosensory function in *C. elegans*.

## Discussion

The importance of the ubiquitin system for controlling diverse features of neuronal function is well established (Kowalski & Juo, 2012; Tai & Schuman, 2008). Recent studies have implicated the USS in controlling aspects of sensory system function in both *C. elegans* and other species (Chen & Chalfie, 2015; Gierthmuhlen et al., 2010; James, Key & Beverdam, 2014; Kim et al., 2015; Lu et al., 2009; Sourisseau et al., 2001). The APC ubiquitin ligase is a known regulator of many of these processes, including neuronal survival, axon outgrowth and dendritogenesis, as well as synapse formation and signaling (Kowalski et al., 2014; Puram & Bonni, 2011). In this study, we found that both *emb-27(g48)* and *emb-27(ye143)* loss of function APC mutants exhibited increased chemotaxis to both pyrazine and diacetyl, but chemotaxed normally towards isoamyl alcohol (Fig. 2). We also found that the *emb-27(g48)* mutants showed increased chemotaxis to pyrazine at multiple concentrations over a 2 h time period (Fig. 3). These results support our hypothesis that the APC subunit protein EMB-27 APC6 plays a role in chemosensation towards pyrazine and diacetyl.

When we tested our hypothesis that multiple APC subunits would affect chemosensation, as seen for the APC’s regulation of both glutamatergic and GABAergic transmission in *C. elegans* (Juo & Kaplan, 2004; Kowalski et al., 2014), however, we saw unexpected results. The *mat-2(ax102)* loss of function mutants only exhibited increased chemotaxis to pyrazine, but not to diacetyl, relative to the wild type controls (Fig. 4). Although somewhat surprising, there are several possible explanations for this result. First, it is possible that the *mat-2(ax102*) mutants simply carry a weaker temperature sensitive allele than either of the *emb-27 APC6* mutants. Slightly weaker phenotypes were seen for the effects of *mat-2 (ax102)* on both GLR-1 receptor abundance (Juo & Kaplan, 2004) and NMJ signaling (Kowalski et al., 2014). Alternatively, it is possible that not all subunits of the APC affect chemosensation in the same way, and different APC sub-complexes might function in different contexts or cell types. APC sub-complexes have been isolated from HeLa cells, supporting the potential existence of such sub-complexes in other cell types (Vodermaier et al., 2003); however, despite more complete structural data generated by a number of laboratories (Chang et al., 2014; Yamaguchi et al., 2015), much remains unknown about the timing of complex assembly or even the possibility of ubiquitin-independent sub-complex functions *in vivo*. Future work will be needed to fully explore these questions. This study supports the idea that these sub-complexes may exist, as a sub-complex of the APC including the EMB-27 APC6 protein but possibly not MAT-2 APC1 may impact chemotaxis to certain odorants.

Because the *emb-27 APC6* mutant worms showed normal responses to some odorants and increased chemotaxis to others, it can be posited that the effect of the APC on chemosensation may be due to effects in specific sensory neurons rather than to the function of the APC in motor neurons or interneurons. Worms lacking AWC chemosensory neurons have defective responses toward isoamyl alcohol, and worms lacking AWA chemosensory neurons have defective responses toward diacetyl and pyrazine (Bargmann, Hartwieg & Horvitz, 1993). Because the *emb-27 APC6* mutants were defective to diacetyl and pyrazine but not to isoamyl alcohol, we hypothesize that the APC inhibits chemotaxis toward pyrazine and diacetyl by controlling signaling events downstream of receptors for both of these molecules in the AWA sensory neurons. It is also possible that rather than defects on intracellular signaling and responses, loss of APC function leads to altered AWA cilia morphology or cell fate. Future studies investigating the expression pattern of various APC subunits, as well as structural studies aimed at visualizing cilia morphology will be required to further explore this possibility.

If the APC does act in AWA neurons, as the simplest model would suggest, what are the potential targets of the APC in these cells? The APC typically adds K11 linked ubiquitin chains to its target proteins (Jin et al., 2008; Williamson et al., 2011; Wu et al., 2010); this particular branching pattern has been shown to target proteins for proteasomal degradation (Kulathu & Komander, 2012). Given that the APC typically promotes degradation of its substrates (Peters, 2006), we hypothesize that the APC similarly acts to negatively regulate the abundance of its target(s) in olfactory signaling. Based on this, we predict that the loss of function of an APC substrate protein in this context would cause decreased responsiveness to AWA odorants. Although much remains unknown about the signaling events that occur downstream of odorant receptor activation, several possible candidates exist for mediating the effects of the APC on AWA olfactory function. These include the nuclear hormone receptor ODR-7, which is required to promote AWA neuron cell fate and to repress some aspects of AWC fate (Sagasti et al., 1999; Sengupta, Chou & Bargmann, 1996; Sengupta, Colbert & Bargmann, 1994), as well as members of the TRPV ion channel heterodimer, OSM-9 and OCR-2 (Colbert, Smith & Bargmann, 1997; Sengupta, Colbert & Bargmann, 1994; Tobin et al., 2002). Interestingly, the requirement for *odr-7* in repressing AWC-specific gene expression may explain the slight increase in chemotaxis to the AWC odorant isoamyl alcohol we observed in *odr-7* mutants (Figs. 2 and 4).

Despite the AWA-specific functions of ODR-7, the OSM-9/OCR-2 channel represents the most likely cell autonomous target of the APC. Like *odr-*7, the TRPV channel genes, *osm-9* and *ocr-2* are expressed together in AWA and in several other sensory neuron classes, and, although the *osm-9* gene is also expressed in AWC neurons, both *osm-9* and *ocr-2* are required for chemotaxis to AWA-specific odorants but not to chemicals sensed by AWC neurons (Colbert, Smith & Bargmann, 1997; Tobin et al., 2002). Together, the OSM-9 and OCR-2 proteins act as a channel complex that is thought to be opened downstream of the protein kinase C (nPKC) epsilon enzyme TTX-4 (Okochi et al., 2005). TTX-4 is activated following odorant-receptor binding and subsequent G-protein signaling via the $\mathrm{G}\alpha $ protein ODR-3, among others, in AWA neurons (Bargmann, 2006; Roayaie et al., 1998; Zhang et al., 2014). OSM-9 and OCR-2 are reciprocally required for one another’s ciliary localization, and the OCR-2 protein, like the other OCR family members (OCR1-4), contains a D-box motif (amino acids 282-290, RLL LAFKA N) in its N-terminal cytoplasmic tail. One possibility is that the APC negatively regulates AWA chemosensory function by promoting the ubiquitination and degradation of OCR-2, which would in turn prevent proper function and localization of the OSM-9/OCR-2 channel and thus inhibit changes in gene expression and/or membrane potential needed for neurotransmitter release from the AWA neurons.

While OCR-2 represents one known potential target of the APC in AWA neurons, there are likely other candidates; these may include AWA-specific genes like ODR-7 (Sengupta, Colbert & Bargmann, 1994), genes known to function in multiple neuron classes, such as ODR-3 (Roayaie et al., 1998), which may be differentially regulated by the APC in AWA neurons or in other cells to impact AWA function, as well as additional novel targets that have yet to be described. Future studies aimed at testing specific known candidate substrates of the APC or screening for novel APC targets in this context are required to fully elucidate the mechanisms by which this enzyme contributes to olfactory regulation. Additional cell type-specific rescue and expression studies assessing possible contributions of APC function in not only sensory neurons, but also interneurons activated downstream of AWA, such as the AIY, AIA or AIZ interneurons (Taniguchi et al., 2014; Tsalik & Hobert, 2003; White et al., 1986), should also be explored, as the APC could also contribute to differential AWA sensory circuits at this level.

## Conclusions

The APC plays diverse roles in neuronal physiology (Puram & Bonni, 2011), including critical functions in controlling synaptic transmission at glutamatergic synapses (Juo & Kaplan, 2004) and in regulating GABAergic signaling to control excitatory to inhibitory balance at the neuromuscular junction (Kowalski et al., 2014). Here, we demonstrated that the APC regulates chemosensation, as worms lacking function of the APC protein EMB-27 APC6 exhibited increased chemotaxis specifically to odorants sensed by AWA neurons but not to an AWC-specific odorant, leading us to conclude that the APC may function in sensory neurons, in addition to its previously described roles in interneurons and motor neurons. This is the first study to examine the effect of the APC on sensory function in *C. elegans* or in any system. Additionally, we have seen that not all subunits of the APC may affect chemosensation equally in *C. elegans*; this may indicate that a specific sub-complex of the APC controls this behavior. This and future investigations aimed at identifying potential APC substrates and regulators relevant in this context, the APC’s ability to act in specific classes of sensory neurons, as well as additional sensory functions in which the APC may be involved (e.g., attraction or repulsion to water-soluble cues, thermosensation, mechanosensation, and sensory adaptation), will lead to a greater understanding of the complicated nature of this protein complex and its potential uncharacterized roles in sensory biology. Given the conservation of the APC and sensory system structure and function across phylogeny, this information may have implications for understanding human sensory and ubiquitin enzyme function.

## Supplemental Information

10.7717/peerj.2013/supp-1 Data S1 Chemotaxis Raw Data

## Additional Information and Declarations

### Data Availability

The following information was supplied regarding data availability: The raw data has been supplied as Data S1.
